# Exploration of a Robust and Prognostic Immune Related Gene Signature for Cervical Squamous Cell Carcinoma

**DOI:** 10.3389/fmolb.2021.625470

**Published:** 2021-03-03

**Authors:** Zhihua Zuo, Junjun Xiong, Chuyi Zeng, Yao Jiang, Kang Xiong, Hualin Tao, Yongcan Guo

**Affiliations:** ^1^ Department of Clinical Laboratory, The Affiliated Hospital of Southwest Medical University, Luzhou, China; ^2^ Department of Gynaecology, The Affiliated Hospital of Southwest Medical University, Luzhou, China; ^3^ Department of Oncology, The Affiliated Hospital of Southwest Medical University, Luzhou, China; ^4^ Department of Clinical Laboratory, Traditional Chinese Medicine Hospital Affiliated to Southwest Medical University, Luzhou, China

**Keywords:** cervical squamous cell carcinoma, weighted gene co-expression network analysis, immune cells infiltration, prognosis, immunotherapy sensitivity

## Abstract

**Background:** Cervical squamous cell carcinoma (CESC) is one of the most frequent malignancies in women worldwide. The level of immune cell infiltration and immune-related genes (IRGs) can significantly affect the prognosis and immunotherapy of CESC patients. Thus, this study aimed to identify an immune-related prognostic signature for CESC.

**Methods:** TCGA-CESC cohorts, obtained from TCGA database, were divided into the training group and testing group; while GSE44001 dataset from GEO database was viewed as external validation group. ESTIMATE algorithm was applied to evaluate the infiltration levels of immune cells of CESC patients. IRGs were screened out through weighted gene co-expression network analysis (WGCNA). A multi-gene prognostic signature based on IRGs was constructed using LASSO penalized Cox proportional hazards regression, which was validated through Kaplan–Meier, Cox, and receiver operating characteristic curve (ROC) analyses. The abundance of immune cells was calculated using ssGSEA algorithm in the ImmuCellAI database, and the response to immunotherapy was evaluated using immunophenoscore (IPS) analysis and the TIDE algorithm.

**Results:** In TCGA-CESC cohorts, higher levels of immune cell infiltration were closely associated with better prognoses. Moreover, a prognostic signature was constructed using three IRGs. Based on this given signature, Kaplan–Meier analysis suggested the significant differences in overall survival (OS) and the ROC analysis demonstrated its robust predictive potential for CESC prognosis, further confirmed by internal and external validation. Additionally, multivariate Cox analysis revealed that the three IRGs signature served as an independent prognostic factor for CESC. In the three-IRGs signature low-risk group, the infiltrating immune cells (B cells, CD4/8 + T cells, cytotoxic T cells, macrophages and so on) were much more abundant than that in high-risk group. Ultimately, IPS and TIDE analyses showed that low-risk CESC patients appeared to present with a better response to immunotherapy and a better prognosis than high-risk patients.

**Conclusion:** The present prognostic signature based on three IRGs (CD3E, CD3D, LCK) was not only reliable for survival prediction but efficient to predict the clinical response to immunotherapy for CESC patients, which might assist in guiding more precise individual treatment in the future.

## Introduction

Cervical cancer ranks as the fourth most frequent malignancy and the fourth leading cause of cancer-related death in women worldwide, with approximately 570,000 new cases and 311,000 deaths reported by the International Agency for Research on Cancer (IARC) in 2018 alone (Bray et al., 2018). Cervical squamous cell carcinoma (CESC) comprises the primary histological type of cervical cancer, accounting for more than 80% of cases. Although recent advances in comprehensive treatments, including surgical resection, chemotherapy, and radiotherapy, have been made, the overall mortality has increased annually around the world (Jemal et al., 2011; Torre et al., 2015; Bray et al., 2018). For instance, in China, the number of deaths increased at an annual percentage change of 5.9% from 2001 to 2011 (Chen et al., 2016). Notably, the high risk of metastasis and recurrence makes it challenging for CESC patients with traditional therapies to maintain a good prognosis; thus, the development of a reliable prognostic assessment and novel therapeutic strategies is urgently needed (Pfaendler and Tewari, 2016).

In recent years, immunotherapy, especially the use of immune checkpoint inhibitors (ICIs), has made appreciable progress in antitumor practice and is becoming the new first-line therapeutic option in oncology (Fillon, 2020); e.g., for treatment of bladder cancer (Patel et al., 2020), melanoma (Zaretsky et al., 2016), renal cancer (Curti, 2018). Distinct from traditional treatments, the clinical benefits for immunotherapy patients are achieved by stimulating the persistent antitumor immune response (Kennedy and Salama, 2020), which depends on immunomodulation between the tumor microenvironment (TME) and cancer cells. Numerous studies have reported that infiltrating immune cells, a vital part of the TME, are closely related to cancer progression and the efficacy of immunotherapy (Hanahan and Coussens, 2012; Byrne et al., 2020). For instance, the mechanism of immune checkpoint blockade (ICB) therapy is to enhance a patient’s anti-tumor immune response by blocking the inhibitory effect of tumor cells on immune cells. In this process, programmed cell death 1 (PD1) and cytotoxic T lymphocyte antigen 4 (CTLA-4) are the most common targets of ICIs, which generally inhibit the activation and amplification of T cells that render the anti-tumor response ineffective. Although ICIs were expected to exhibit great potential in the immunotherapy of CESC patients, the clinical outcomes and prognosis were far from satisfactory, as only less than 20% patients achieved a partial or complete response where most had a PD-L1-positive tumors (Frenel et al., 2017; Chung et al., 2019). Meanwhile, the heterogeneity of increased levels of tumor infiltrating lymphocytes and tumor mutational burden in CESC participants also gives the additional explanation for it (Otter et al., 2019). Therefore, to achieve precise personalized decision-making for ICI treatment, a robust prognostic signature will be important to determine the prognosis and predict the sensitivity of immunotherapy in CESC patients.

Although several risk models have been developed to predict the prognosis of CESC patients (Yang et al., 2019; Liu et al., 2020), these have been based on differentially expressed genes, which ignored the complicated interactions of genes, as well as hub genes that showed no significant differences between tumor and normal tissues, but were closely linked to clinical features. Additionally, a promising prognostic signature based on immune-related genes (IRGs) has been confirmed great potential to predict prognosis and immunotherapy responsiveness in cancers but were less reported in CESC. In the present study, weighted gene co-expression network analysis (WGCNA) was the first time to be applied to identify significant IRGs for CESC patients, which were highly associated with infiltration levels of immune cells calculated by the use of ESTIMATE algorithm. Furthermore, hub IRGs were selected to construct a promising prognostic signature through the least absolute shrinkage and selection operator (LASSO) penalized Cox proportional hazards regression. Ultimately, internal and external validations further demonstrated that this robust prognostic signature based on IRGs in this study was not only reliable for survival prediction but efficient to predict the clinical response of ICIs for CESC patients, which might facilitate personalized counseling for immunotherapy in the future.

## Methods

### Data Acquisition and Preprocessing

The RNA-seq datasets (FPKM profiles) and clinical information of the CESC cohorts were retrieved from The Cancer Genome Atlas (TCGA) website (https://portal.gdc.cancer.gov/). Then, the gene expression matrix was formed via Perl script based on the CMD command. IDs were converted to gene symbols according to the Ensembl database (http://asia.ensembl.org/index.html). Meanwhile, mRNA expression profiles (GSE44001) were also downloaded from Gene Expression Omnibus (GEO) database to be viewed as the external validation group, where the matrix profile was conducted by Robust Multi-Array Average (RMA) algorithm in R affyPLM package (Irizarry et al., 2003). But of note, any patients with the follow-up time less than 1 day were excluded. In addition, if matched with multiple IDs in two matrix profiles, the gene expression values were averaged. Finally, log2 processing of the matrix data was performed for further analyses.

### Evaluation of Tumor Microenvironment in The Cancer Genome Atlas-Cervical Squamous Cell Carcinoma Cohort

ESTIMATE algorithm (Yoshihara et al., 2013) has been popularly applied to predict infiltration levels of immune cells and stromal cells in the TME based on specific gene expression, of which the final results were evaluated using the immune score and stromal score, respectively. The ESTIMATE score was also calculated to measure the overall tumor immunity of CESC. Then, all patients were divided into two groups (high score and low score) based on the mean score, and survival analyses were performed to explore whether the overall survival were related with the immune infiltration levels.

### Construction of Weighted Gene Co-expression Networks

In the present study, the top 25% most-varying genes calculated by the mean absolute deviation (MAD) algorithm were selected for subsequent analysis. The outlier samples were firstly identified and removed using the goodSamplesGenes function in the WGCNA package. Next, Pearson correlation coefficients were calculated between any two genes to construct the gene expression similarity matrix with the following formula: ${\text{a}}_{\text{ij}}=\;{\left|\text{cor }\left({\text{X}}_{\text{i}}{\text{, X}}_{\text{j}}\right)\right|}^{\text{β}}$, where X_i_ and X_j_ represent the expression values of gene i and j. Furthermore, the cut-off value of scale-free R2 was set at 0.9 to obtain the lowest soft-thresholding power β that would be used to build an adjacency matrix so that gene distribution conformed to scale-free networks. Based on the topological overlap matrix (TOM) converted from the adjacency matrix, dissimilarity between genes was employed to cluster genes with similar expression profiles by the hierarchical clustering method, which were then cut into different modules by dynamic shear module recognition and visualized by the dendrogram with colored assignments (minimum module size was 80). Meanwhile, a cut-height of 0.35 was considered as the cut-off value to merge similar modules.

### Identification of Significant Modules and Genes Related to Clinical Features

The clinical traits of each sample were combined with module eigengenes (MEs) to construct the relevant clinical modules. MEs were defined as the first principal component of the module, representing the gene expression profile of the entire module. The clustering modules that were the most closely associated with immune cell infiltration were selected as the significant modules. Gene significance (GS) and module membership (MM) were also calculated to screen significant genes. GS represents the correlation between gene expression and clinical traits, and MM reflects the correlation between gene expression profile and genes within given modules. Ultimately, GS > 0.4 and MM > 0.8 were set as the criteria to identify hub genes that were strongly related to the clinically significant traits. (Zhang and Horvath, 2005).

To further screen potential genes that play an essential role in immune cell infiltration of CESC, a total of 1811 IRGs were obtained from the ImmPort database (https://immport.niaid.nih.gov) (Bhattacharya et al., 2014), and these overlapping genes were selected as hub IRGs for subsequent analysis.

### Functional Enrichment and Interactions Analyses

The functional enrichment analyses including Gene Ontology (GO) enrichment and Kyoto Encyclopedia of Genes and Genomes (KEGG) pathway analyses were performed using the R clusterProfiler package (Yu et al., 2012). GO terms were further divided into biological process (BP), molecular function (MF), and cellular component (CC). Adj. *p*. value <0.05 were considered significant. To identify the interactive relationships of hub genes, a protein–protein interaction (PPI) network was constructed using the STRING database (https://string-db.org/) with the minimum required interaction score being 0.7. In addition, the R corrplot package was applied to conduct Pearson correlation analysis between hub IRGs.

### Development and Validation of the Prognostic Signature

CESC patients were randomly divided into training and test groups with a 7:3 ratio (Table 1). In the training set, univariate Cox regression analysis was performed to explore the relationship between the expression of each key IRG and overall survival (OS). Then, these genes with a *p*-value < 0.05 were selected as hub IRGs, which were further analyzed by using LASSO penalized Cox proportional hazards regression to find the best risk model in the R package “glmnet” (Wang et al., 2019a). The risk score was calculated using the following formula: $risk\;score=\left(\beta_{1}*G_{1}+\beta_{2}*G_{2}+\beta_{3}*G_{3}+\cdots +\beta_{n}*G_{n}\right)$, where *β* is the coefficient of each prognostic hub gene, *G* represents the expression value of hub genes, and *n* denotes the number of hub genes. Patients were classified into low-risk (<mean risk score) and high-risk (>mean risk score) groups depending on the mean risk score. Moreover, the survival curve was determined using the Kaplan–Meier method in the R survminer package, where the differences between low- and high-risk groups were calculated by the log-rank test. Meanwhile, a time-dependent receiver operating characteristic (ROC) curve was adopted using R ROC package (Kamarudin et al., 2017), of which the area under the curve (AUC) was calculated to assess the accuracy of the prognostic risk model.

**TABLE 1:** Clinical variables in the training and testing sets.

Characteristic	Total n = 288	Training set n = 201	Testing set n = 87	*p* Value
Age	48.2 ± 13.8	47.7 ± 14.1	49.4 ± 13.17	0.4331
Race				0.9887
African	30	21	9	
Asian	28	18	10	
White	201	140	61	
Unknown	29	22	7	
FIGO stage				0.7656
Ⅰ	156	104	52	
Ⅱ	64	51	13	
Ⅲ	41	29	12	
Ⅳ	21	14	7	
Unknown	6	3	3	
Stromal score (min, max)	(−2,586.99, 778.01)	(−2,586.99, 778.01)	(−2,400.89, 451.87)	>0.9999
Immune score (min, max)	(−1,645.63, 3,295.3)	(−1,645.63, 2,651.87)	(−1,356.39, 3,295.3)	0.8698
ESTIMATE score (min, max)	(−3,643.23, 3,744.09)	(−3,643.23, 1995.06)	(−3,234.1, 3,744.09)	0.8917

FIGO stage, International Federation of Gynecology and Obstetrics stage.

To further verify the predictive performance of the prognostic model, the risk scores were also calculated in the testing group and external groups using the same prognostic formula, and the Kaplan–Meier survival curve and ROC curve were generated with a cutoff value of the mean risk score.

### Immune Infiltration Patterns with Prognostic Signature

Immune infiltrating cells, such as T cells, B cells, and monocytes, have been shown to be important in the TME and to significantly affect cancer progression. Thus, ImmuCellAI (http://bioinfo.life.hust.edu.cn/web/ImmuCellAI/) (Miao et al., 2020), a novel and efficient tool based on the ssGSEA algorithm that can estimate the abundance of 24 immune infiltrate cells (18 T-cell subtypes and B cells, natural killer cells, monocytes, macrophages, neutrophils, and dendritic cells) from the gene expression data set (Bindea et al., 2013), was applied to calculate the abundance of immune cells and to compare the differences in infiltrated patterns between low- and high-risk groups. Meanwhile, we also employed TIMER database to explore the correlation of the expression levels of hub IRGs with the expression of immune checkpoint molecules and infiltrating levels of immune cells (Li et al., 2017).

### Immunogenicity and Immunotherapeutic Sensitivity with Prognostic Signature

To further validate the predictive performance of the given prognostic signature for the response to ICIs, two independent methods including immunophenoscore (IPS) analysis and the Tumor Immune Dysfunction and Exclusion (TIDE) algorithm were employed to assess the immunogenicity and immunotherapeutic sensitivity of CESC patients, respectively.

The immunogenicity of a patient was determined by four main parts (effector cells, immunosuppressive cells, MHC molecules, and immunomodulators), which could be calculated without bias using machine learning methods by IPS analysis (Charoentong et al., 2017). Higher IPS scores (range 0–10) represent increased immunogenicity. The IPSs of patients with CESC were obtained from The Cancer Immunome Atlas (TCIA) (https://tcia.at/home).

The TIDE algorithm, designed by Dr. Shirley Liu and colleagues, was considered as highly reliable method to predict the clinical response of patients to ICB therapy (PD1 and CTLA-4 inhibitor) in recent studies (Jiang et al., 2018). The results were measured by TIDE score. According to the default settings, a patient with a TIDE value <0 was defined as a responder (positive sensitivity to immunotherapy), whereas a patient with a TIDE value >0 was defined as a non-responder (negative sensitivity to immunotherapy). Furthermore, a correlation analysis of CESC samples was performed to explore the correlation between the given prognostic signature and immune function.

### Statistical Analysis

The WGCNA method and all statistical analyses were performed in R software (3.6.1) and GraphPad Prism (8.0). The Mann–Whitney test was applied to compare differences of continuous data between two groups, whereas ANOVA was used for more than two groups. A Chi square test was used to test for differences between categorical variables. *p* values <0.05 were considered to be statistically significant.

## Results

### Correlation of ESTIMATE Score and Clinical Characteristics

**FIGURE 1:**
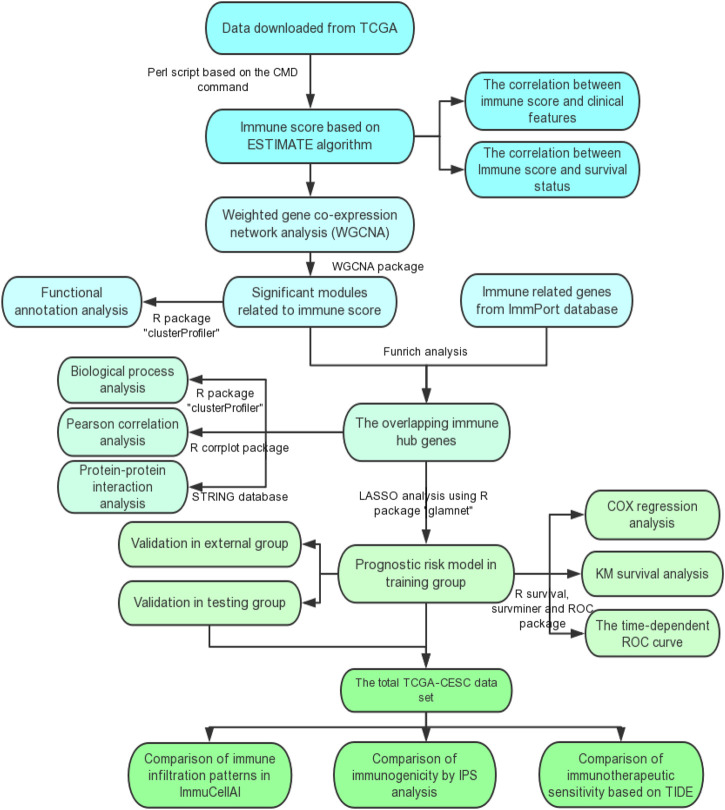
The flow diagram of this study. TCGA, the Cancer Genome Atlas; GO, Gene Ontology; KEGG, Kyoto Encyclopedia of Genes and Genomes; ROC curve, receiver operating characteristic curve; IPS, Immunophenoscore; TIDE, the Tumor Immune Dysfunction and Exclusion.

**FIGURE 2:**
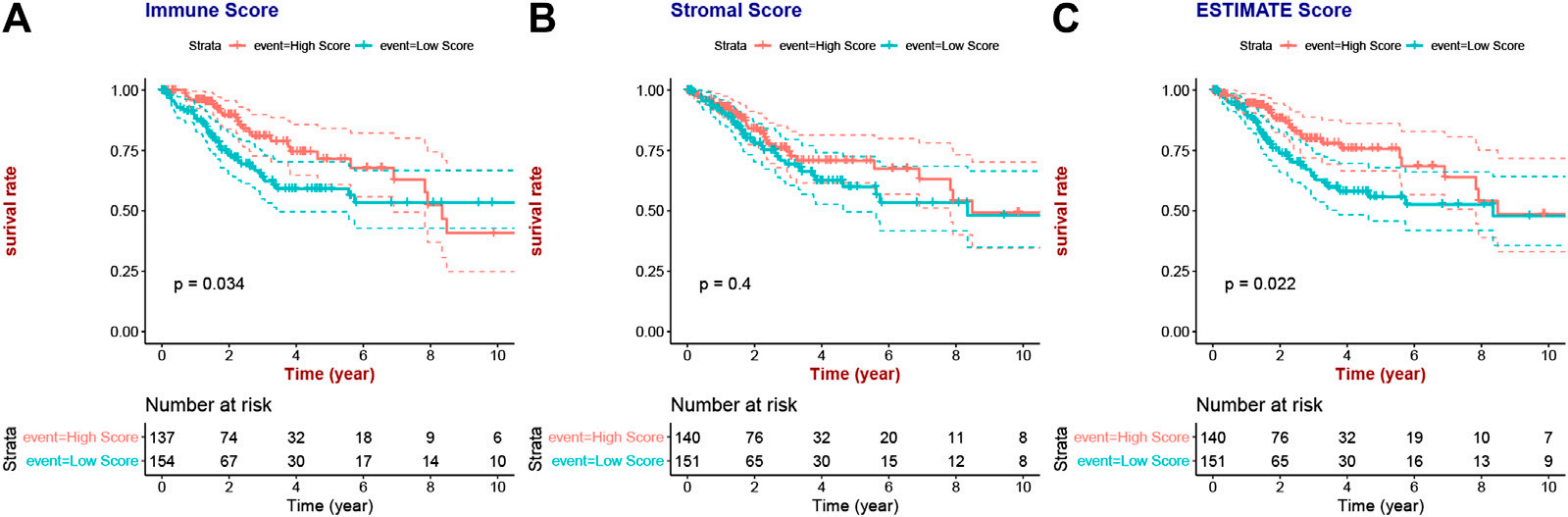
Associations between immune/stromal/ESTIMATE scores and CESC patients’ prognosis.

The flow diagram of the present study is shown in Figure 1. A total of 291 and 301 CESC samples were enrolled from TCGA and GEO database according to the inclusion criteria, respectively. The ESTIMATE score of each patient, which reflects the landscape of the TME and overall immune-infiltration degree was calculated with the ESTIMATE algorithm. As shown in Figure 2, patients with high immune and ESTIMATE scores demonstrated a better OS than those with low scores (*p* < 0.05). Nevertheless, all scores showed no statistical significance between different race, age, and International Federation of Gynecology and Obstetrics (FIGO) stage (Supplementary Figure S1 and Supplementary Table S1).

### Identification of Immune-Related Modules and Hub Genes by Weighted Gene Co-Expression Network Analysis

**FIGURE 3:**
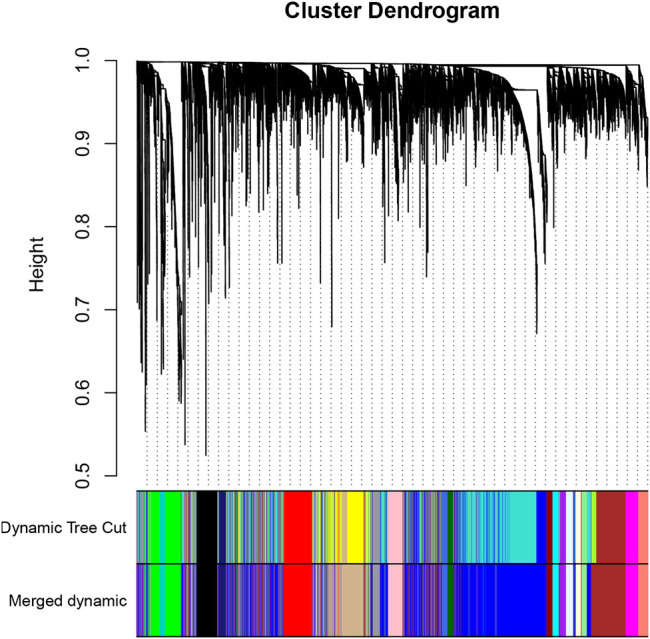
The cluster dendrogram of module eigengenes.

**FIGURE 4:**
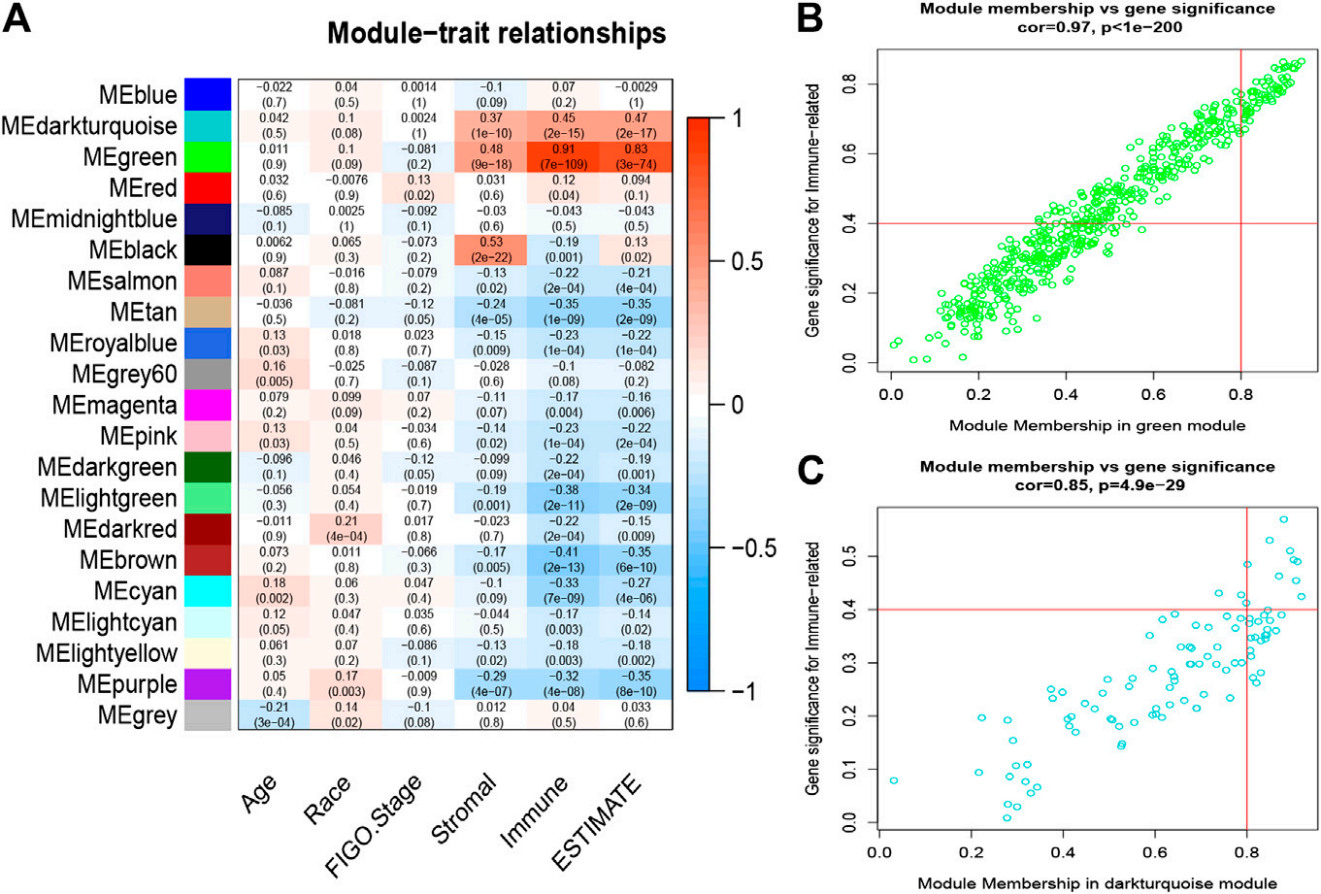
Analysis of key immune-related modules. **(A)**, The correlation between modules and traits was displayed. **(B–C)** The correlation between GS and MM in the green and dark-turquoise modules. GS, gene significance; MM, module membership.

First, three outlier samples were excluded by the goodSamplesGenes function. The dendrogram and heatmap (Supplementary Figure S2) display the distribution and clinical traits of the remaining 288 samples in detail. The soft-threshold power β = 4, the first parameter to meet the requirements of scale-free R2 > 0.9, was selected to construct the scale-free networks (Supplementary Figure S3). Furthermore, after similar modules were merged with a cut-height of 0.35, a total of 21 modules was ultimately identified for further analyses (Figure 3). Interestingly, the green and dark turquoise modules were found to be most associated with immune cell infiltration levels of CESC in the TME (Figure 4A), in which the correlation coefficients were 0.97 and 0.85, respectively. Therefore, the green (Figure 4B) and dark turquoise (Figure 4C) modules were considered immune-related key modules, and significant genes were selected with cut-off criteria of GS > 0.4 and MM > 0.8.

### Function Analyses of Immune-Related Modules and Hub Genes

**FIGURE 5:**
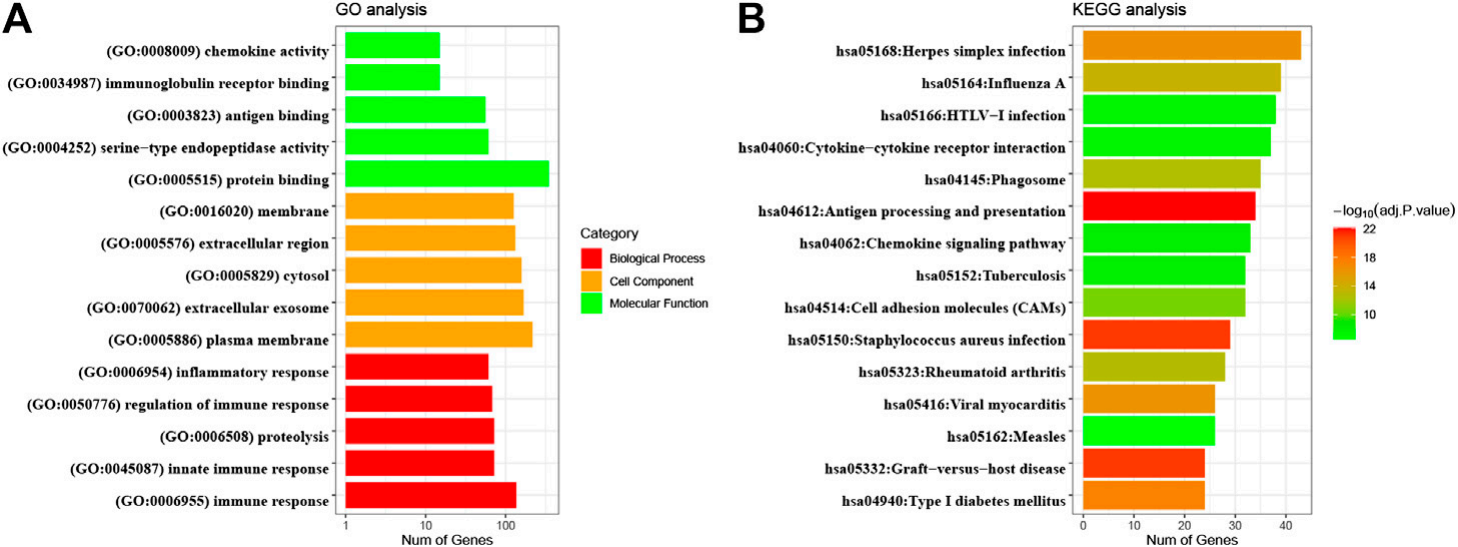
GO enrichment and KEGG pathway analyses of significant module genes. GO, Gene Ontology; KEGG, Kyoto Encyclopedia of Genes and Genomes.

As shown in Figure 5A, most genes within immune-related modules were highly enriched in the inflammatory response, immune response, and proteolysis in the BP category, while chemokine activity, immunoglobulin receptor binding, and antigen binding; and plasma membrane, extracellular exosome, and cytosol were enriched in the MF and CC categories, respectively. Moreover, KEGG pathway analysis (Figure 5B) indicated that virus infection, cytokine−cytokine receptor interaction, antigen processing and presentation, and chemokine signaling pathway were significant. Therefore, the above results suggested that most defined genes in immune modules positively participated in immune-related biological processes in CESC.

### Establishment and Validation of the Prognostic Signature

**FIGURE 6:**
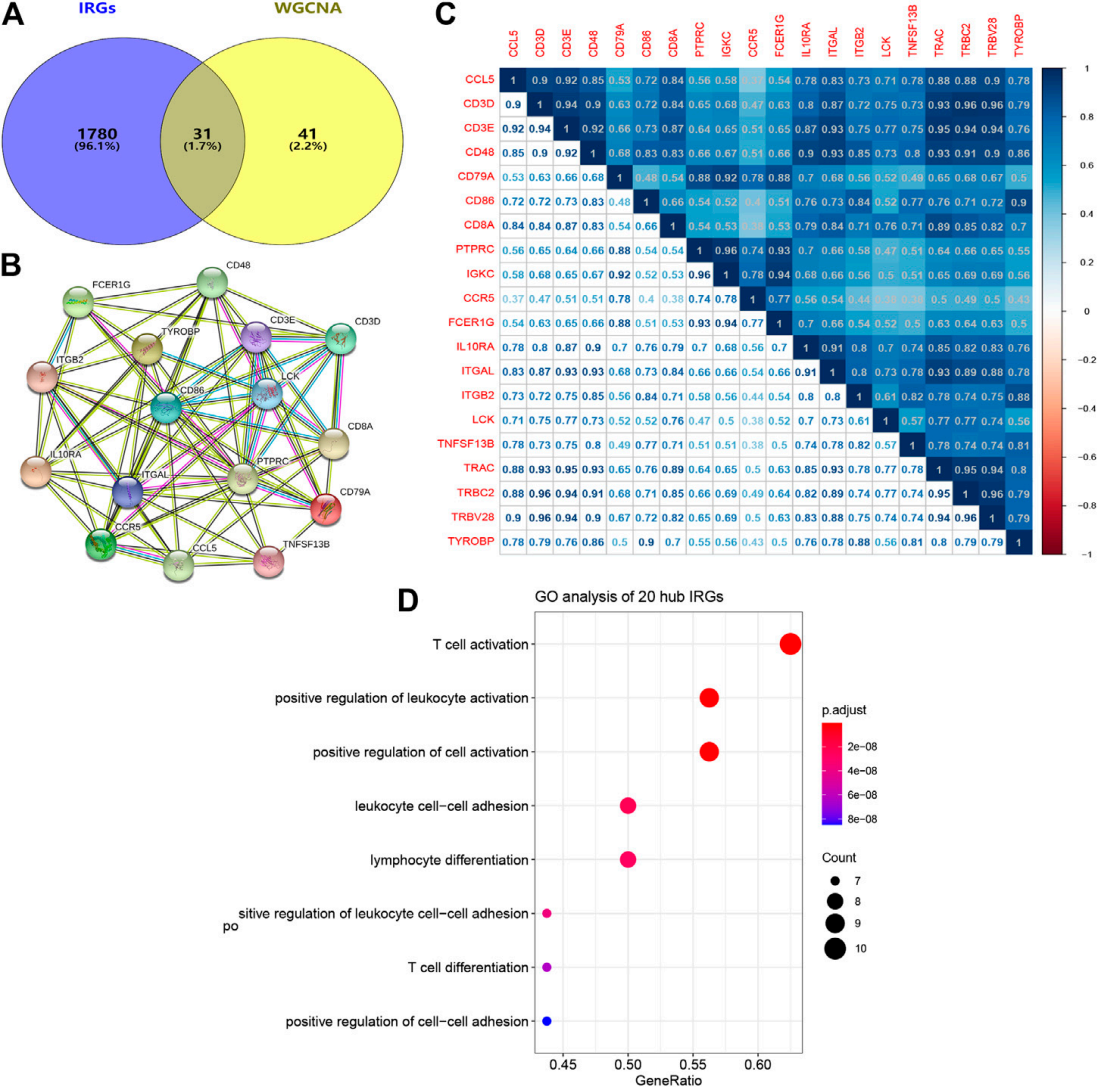
PPI network and biological process analyses of hub IRGs related to prognosis. **(A)** The common genes between modules genes and IRGs from ImmPort database. **(B)** PPI network of 20 hub IRGs. **(C)** Correlation analysis of 20 hub IRGs. **(D)** Biological process analysis of 20 hub IRGs. PPI network, protein-protein interaction network; IRGs, immune-related genes.

A total of 31 common genes were identified between significant module genes and IRGs obtained from the ImmPort database (Figure 6A). Subsequently, TCGA-CESC datasets were randomly divided into a training set (n = 201) and testing set (n = 87); basic clinical information is provided in Table 1. Using univariate Cox regression analysis in the training group, 20 genes were correlated with CESC patient survival (*p* < 0.05) (Table 2). The PPI network is shown in Figure 6B, and the results of Pearson correlation analysis indicated that 20 hub IRGs were significantly co-expressed in CESC samples (Figure 6C). GO analysis (Figure 6D) illustrated that the biological process terms were highly focused on T cell activation, positive regulation of leukocyte activation, leukocyte cell−cell adhesion, lymphocyte differentiation, and T cell differentiation, which further confirmed the important role of identified hub IRGs in the activities of immune infiltration and response.

**TABLE 2:** Identification of 20 immune-related prognostic genes by univariate Cox regression analysis. HR, hazard ratio; CI, Confidence interval.

Gene	Coefficients	HR (95% CI for HR)	Wald.test	*p*. value
TRBV28	−0.296	0.744 (0.634–0.872)	13.3	0.000267
TRBC2	−0.281	0.755 (0.643–0.886)	11.8	0.000581
CD3E	−0.264	0.768 (0.645–0.915)	8.74	0.00311
CD3D	−0.218	0.804 (0.692–0.933)	8.21	0.00416
LCK	−0.31	0.733 (0.587–0.917)	7.41	0.00649
TRAC	−0.235	0.791 (0.666–0.939)	7.2	0.00728
IGKC	−0.138	0.871 (0.787–0.965)	6.98	0.00824
IL10RA	−0.259	0.772 (0.631–0.945)	6.29	0.0121
CD79A	−0.157	0.855 (0.749–0.975)	5.43	0.0197
CCL5	−0.191	0.826 (0.703–0.972)	5.28	0.0216
ITGB2	−0.243	0.785 (0.634–0.972)	4.95	0.0261
CD48	−0.194	0.824 (0.694–0.978)	4.91	0.0266
TNFSF13B	−0.222	0.801 (0.655–0.979)	4.68	0.0305
CCR5	−0.055	0.946 (0.9–0.996)	4.56	0.0328
TYROBP	−0.203	0.816 (0.674–0.988)	4.34	0.0371
CD86	−0.192	0.825 (0.687–0.992)	4.19	0.0405
PTPRC	−0.107	0.898 (0.81–0.997)	4.08	0.0434
ITGAL	−0.169	0.845 (0.716–0.997)	3.99	0.0458
CD8A	−0.157	0.855 (0.733–0.997)	3.98	0.0461
FCER1G	−0.101	0.904 (0.818–0.999)	3.94	0.0473

**FIGURE 7:**
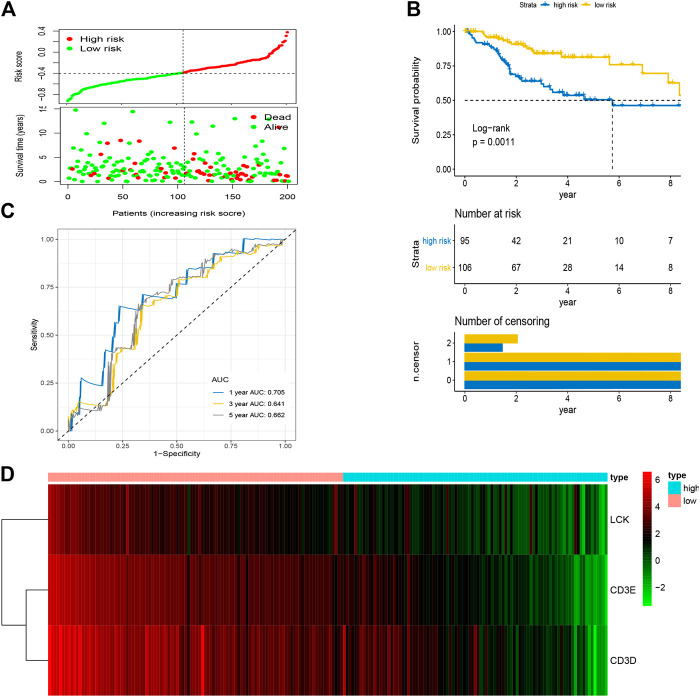
Construction of prognostic signature based on four hub IRGs in training group. **(A)** The distribution of risk scores and survival status between low- and high-risk groups, and mean level of risk score was set as the cut-off value. **(B)** The overall survival analysis of patients in two subgroups. **(C)** ROC curve analysis for the prediction of 1-, 3-, and 5-year OS as the defining point of the four-hub IRGs signature. **(D)** Heatmap of four prognostic IRGs. IRGs, immune-related genes; ROC curve, receiver operating characteristic curve; OS, overall survival.

**FIGURE 8:**
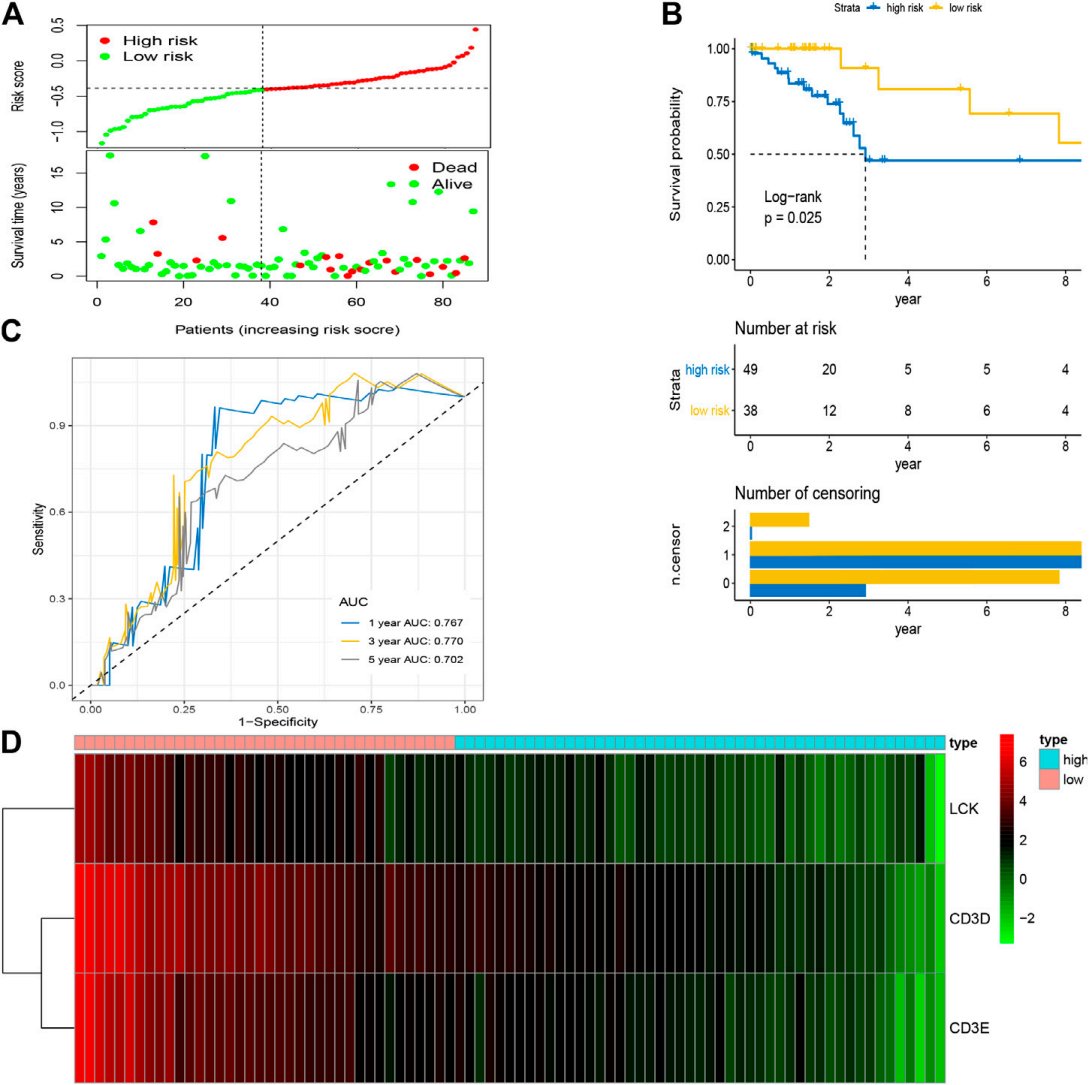
Validation of prognostic signature based on four hub IRGs in testing group. **(A)** The distribution of risk scores and survival status between low- and high-risk groups, and mean level of risk score was set as the cut-off value. **(B)** The overall survival analysis of patients in two subgroups. **(C)** ROC curve analysis for the prediction of 1-, 3-, and 5-year OS as the defining point of the four-hub IRGs signature. **(D)** Heatmap of four prognostic IRGs. IRGs, immune-related genes; ROC curve, receiver operating characteristic curve; OS, overall survival.

**FIGURE 9:**
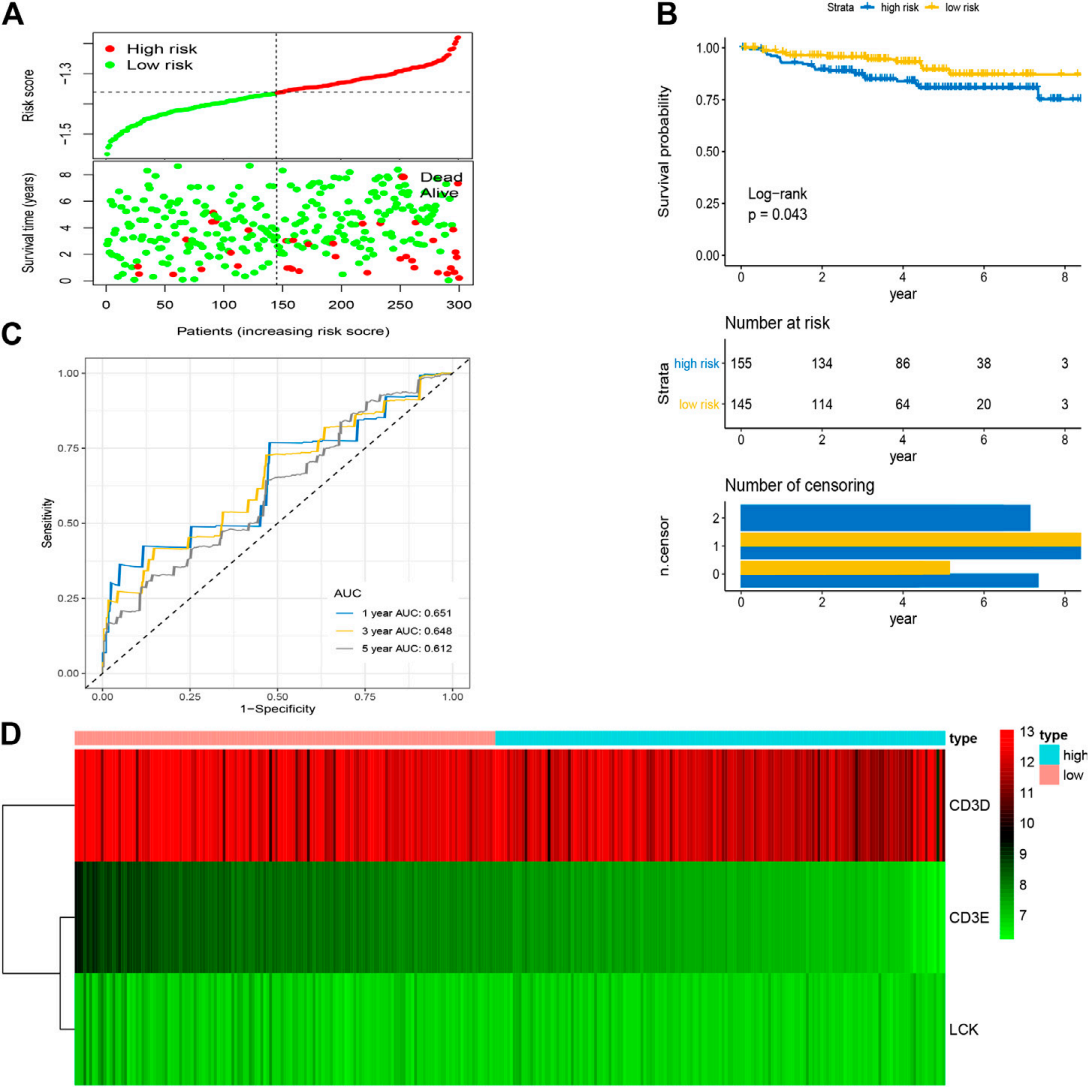
Validation of prognostic signature based on four hub IRGs in external group. **(A)** The distribution of risk scores and survival status between low- and high-risk groups, and mean level of risk score was set as the cut-off value. **(B)** The overall survival analysis of patients in two subgroups. **(C)** ROC curve analysis for the prediction of 1-, 3-, and 5-year OS as the defining point of the four-hub IRGs signature. **(D)** Heatmap of four prognostic IRGs. IRGs, immune-related genes; ROC curve, receiver operating characteristic curve; OS, overall survival.

After the optimal model of prognostic prediction, the individualized risk scores were calculated with coefficient values extracted by LASSO Cox regression analysis. The formula was as follows: risk score = expression of CD3E*(−*0.11337*) + CD3D*(−*0.01026*) + LCK*(−*0.0523*). The LASSO plot is shown in Supplementary Figure S4, and the distribution of patient risk scores and survival status is shown in Figure 7A. The survival analysis indicated that the prognosis of high-risk patients was significantly worse than that of low-risk patients (Figure 7B). Additionally, the time-dependent ROC showed that the AUC values of 1-, 3-, and 5-year OS were 0.705, 0.641, and 0.662, respectively (Figure 7C). Ultimately, the 3-IRGs prognostic signature was validated using OS data from the testing set, of which the results remained consistent (Figure 8D), with the AUC values of 1-, 3-, and 5-year OS of 0.767, 0.770, and 0.702, respectively. Interestingly, the AUC values of 1-, 3-, and 5-year OS were 0.651, 0.648, 0.612 in external group (Figure 9), indicated the reliable predictive potential of the given prognostic signature. Meanwhile, the FIGO stage and prognostic model (low/high) were confirmed as independent risk factors for survival of CESC patients by the univariate and multivariate Cox regression analyses (Table 3).

**TABLE 3:** Univariable and multivariable Cox regression analyses of clinical characteristics.

Variable	Univariate analysis			Multivariate analysis		
	HR	95% CI for HR	P. value	HR	95% CI for HR	P. value
Age	1.02	0.998–1.03	0.084	1.01	0.99–1.03	0.245
FIGO stage	1.49	1.2–1.85	<0.001	1.38	1.11–1.72	0.004
Race	1.01	0.8–1.28	0.927	—	—	—
Risk (low/high)	2.44	1.49–3.99	<0.001	2.24	1.36–3.68	0.002

HR, hazard ratio; CI, Confidence interval; FIGO stage, International Federation of Gynecology and Obstetrics stage.

### Immune Infiltration Patterns with Prognostic Signature

**FIGURE 10:**
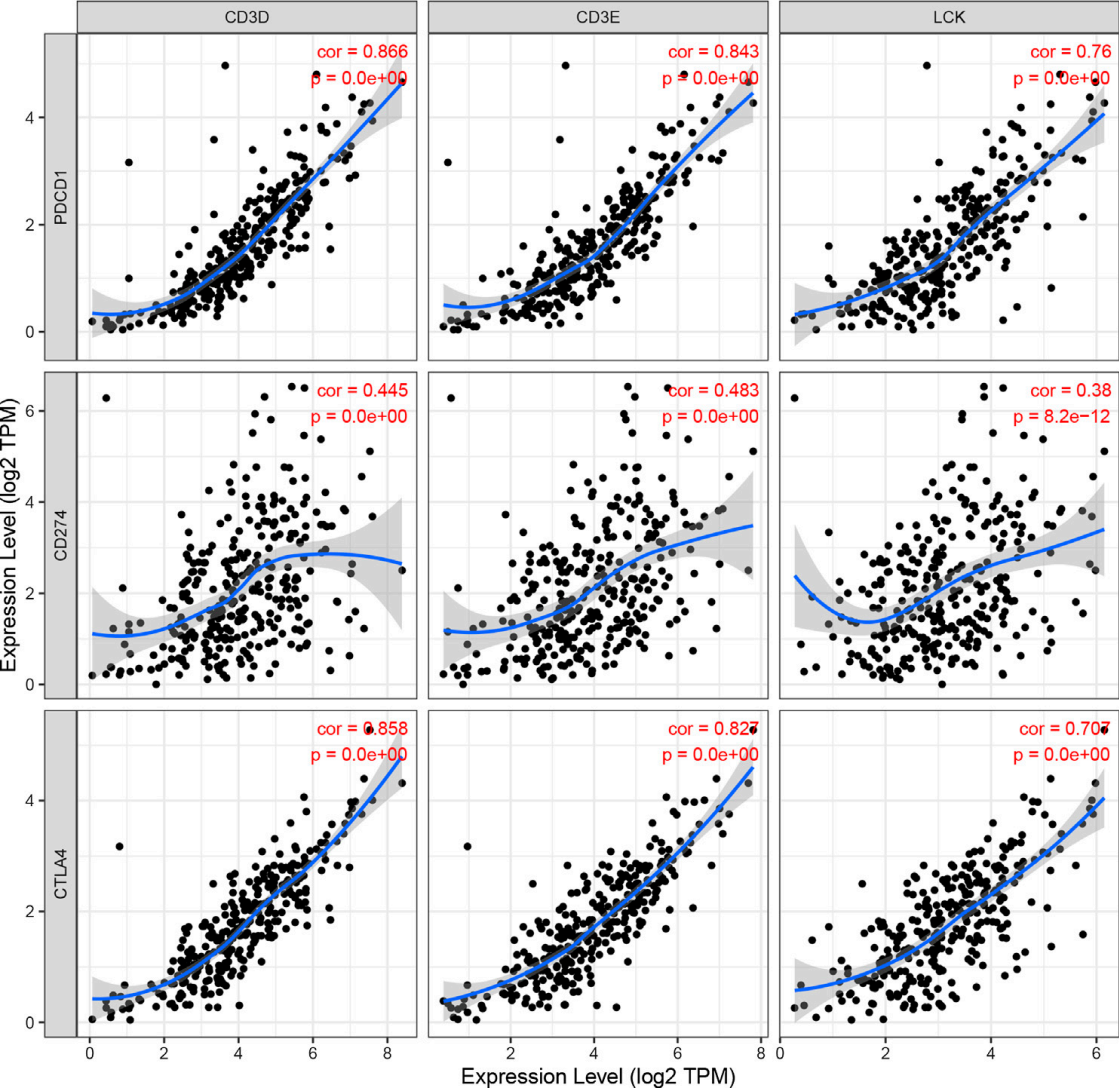
The correlation of CD3D, CD3E, LCK expression with immune checked molecular, including PDCD1(PD1), CD274(PD-L1), and CTLA4.

As shown in Figure 10, the expression levels of CD3D, CD3E and LCK were significantly positively correlation with the expression of three immune checkpoint molecules, including PD1(PDCD1), PDL1(CD274), and CTLA4, respectively. In addition, we also found that the CD3D, CD3E and LCK expression was significantly negatively related to tumor purity while positively associated with the infiltrating levels of immune cells, such as B cell, CD8 + T cell, and CD4 + T cell (Supplementary Figure S5). Thus, these results suggested that CESC patients with higher expression of three IRGs may had a better response to immunotherapy.

**FIGURE 11:**
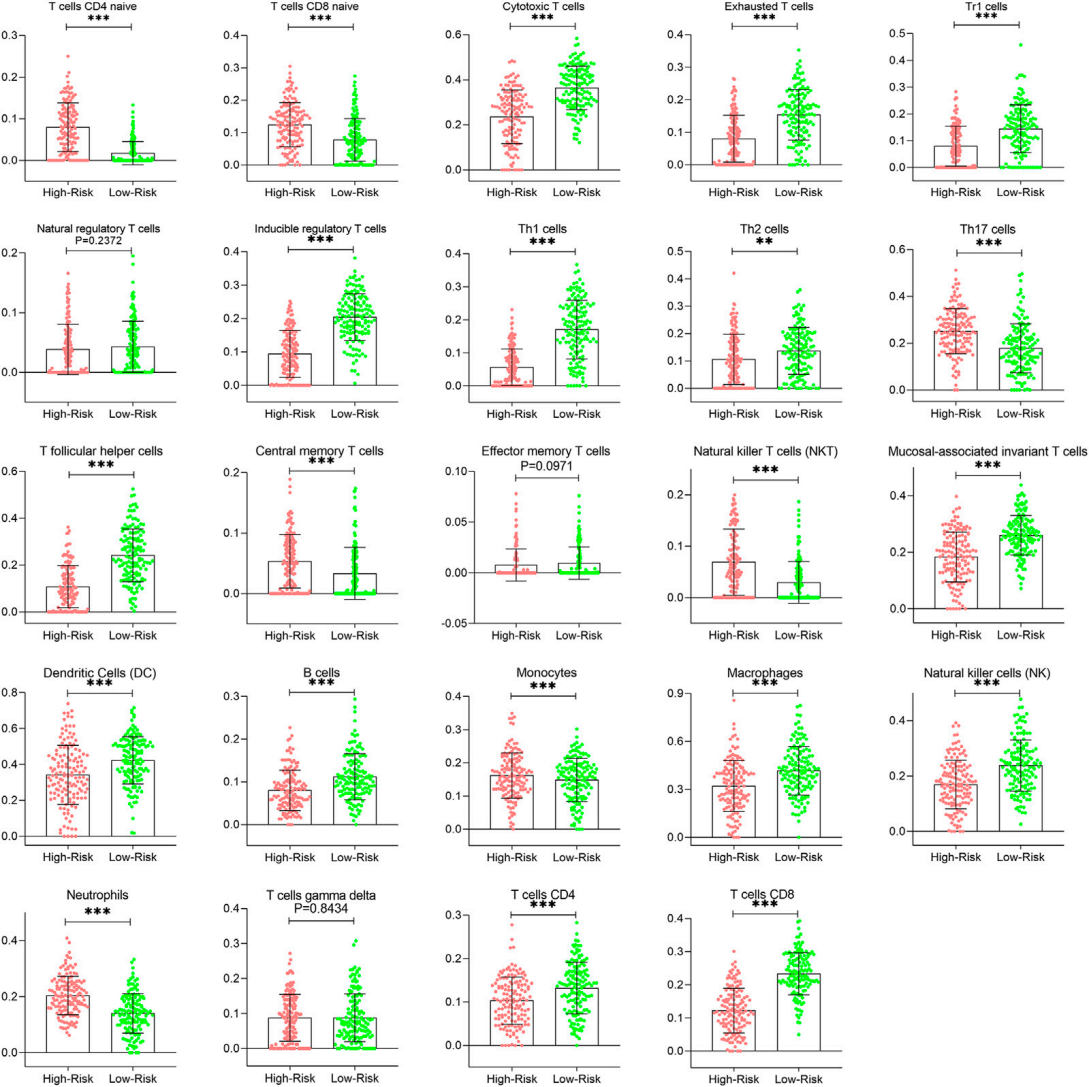
Comparison of immune infiltration patterns of CESC patients between low- and high-risk groups. CESC, cervical squamous cell carcinoma.

The correlation of prognostic signature and ESTIMATE score was shown in Supplementary Figure S6. To further investigate whether there were significant differences in immune cell infiltration between low- and high-risk groups, the abundance of 24 immune cells of each patient was determined using the ssGSEA algorithm in the ImmunCellAI database. As shown in Figure 11, the abundance of immune infiltrating cells, such as CD4/8 naïve T cells, neutrophils, monocytes, and NK T cells, was increased (*p* < 0.05) in the high risk group compared to the low risk group; whereas the infiltrating levels of B cells, CD4/8+ T cells, Th1/2 cells, iTregs, macrophages, and Cytotoxic T cells in the low-risk group were markedly higher than those of the high-risk group. These results are shown in detail in Supplementary Table S2.

### Immunogenicity and Immunotherapeutic Sensitivity with Prognostic Signature

**FIGURE 12:**
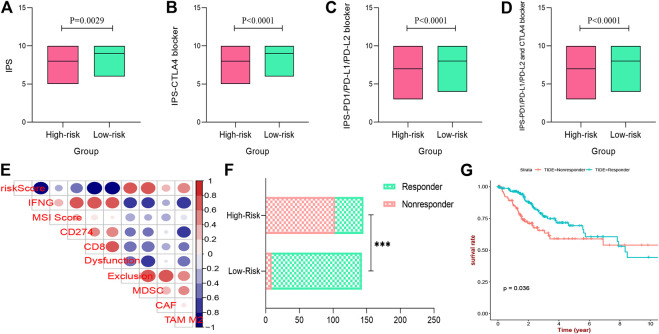
Immunogenicity and immunotherapeutic sensitivity with prognostic signature. **(A–D)** the IPS, IPS-CTLA4 blocker, IPS-PD1-PD-L1-PD-L2 blocker, and IPS-PD1-PD-L1-PD-L2-CTLA4 blocker scores between low- and high-risk groups. **(E)** Immune infiltrating cell profile of tumor microenvironment of CESC patients. **(F)** The differences of immunotherapy sensitivity between low- and high-risk groups. **(G)** Survival analysis of different immunotherapy sensitivity. IPS, Immunophenoscore; ICIs, immune check inhibitors; CESC, cervical squamous cell carcinoma.

In this work, the IPS of each CESC patient was evaluated to explore the association between immunogenicity and the two prognostic risk subgroups. As shown in Figures 12A–D, the IPS, IPS-CTLA4, IPS-PD1-PD-L1-PD-L2, and IPS-PD1-PD-L1-PD-L2-CTLA4 scores in the low-risk group were significantly higher than those of the high-risk group (*p* < 0.05), indicating a more immunogenic phenotype in the low-risk group. In addition, according to the TIDE algorithm, the response to ICIs of CESC patients was measured by the TIDE value (Supplementary Figure S7). The results also indicated that risk scores were significantly negatively correlated with IFNG, CD274 (PD-L1), CD8+ T cells, and dysfunction, and positively correlated with exclusion and MDSC (Figure 12E), which further confirmed our findings; the details are provided in Supplementary Table S3. Moreover, the number of CESC patients that exhibited a positive response to ICIs in the low-risk group was higher than that in the high-risk group (*p* < 0.05) (Figure 12F). The Kaplan–Meier survival curve showed improved survival in the responder group compared with the non-responder group (*p* = 0.036) (Figure 12G). Overall, the results demonstrate that the low-risk group identified by the three IRGs appeared to present with a more positive response to immunotherapy and a better prognosis.

## Discussion

Although advanced diagnostic methods and molecular anticancer therapies have been rapidly developed, the overall prognosis of CESC patients remains poor (McLachlan et al., 2017). ICB therapy was expected to be a breakthrough in CESC treatment (Hamanishi et al., 2017). In several early-phase randomized clinical trials, the application of pembrolizumab and nivolumab (PD-L1/PD-1 blockage) suggested promising clinical outcomes for CESC patients with metastasis and recurrence (Frenel et al., 2017; Chung et al., 2019). However, only a small portion of CESC patients showed a positive response to ICI treatments. Therefore, we identified a robust prognostic signature based on IRGs to forecast prognosis and immunotherapy sensitivity for CESC patients, which may facilitate personalized counseling.

In the present study, the abundance of immune cells and stromal cells in the TME was first investigated using the ESTIMATE algorithm, which was considered a clinical trait for further analysis. The results also indicated that the high infiltration of immune cells showed a better prognosis for CESC patients, indicating that the TME plays an essential role in the prognosis of CESC patients. GO enrichment and KEGG pathway analyses demonstrated that most genes identified as closely related to immune cell infiltration by WGCNA were enriched in immunomodulatory activities, such as the inflammatory response, immune response, and chemokine signaling pathway. By performing univariate and LASSO Cox regression analyses, three hub IRGs (CD3E, CD3D, and LCK) were selected to establish an immune-related prognostic signature for CESC patients, where the AUC values of 1-, 3-, and 5-year OS were 0.705, 0.641, and 0.662 in the training group, and 0.767, 0.770, and 0.702 in the testing group and 0.651, 0.648, 0.612 in the external validation, respectively, indicating the reliably predictive capacity for CESC patient prognosis.

Additionally, functional annotation further suggested that CD3E, CD3D, and LCK were involved in positive regulation of T cell activation and leukocyte cell-cell adhesion that were known as the chief determinant for the efficacy of tumor immunotherapy (Ding and Chen, 2019; Alvarez et al., 2020). Meanwhile, existing evidence has accumulated demonstrating the important role of three hub IRGs in the regulation of immune responses of tumor tissues. The promising tumor immunotherapy mainly depends on the recognition of T-cell receptor (TCR) to special tumor antigens to stimulate the activation of self T cells in order to attack cancer cells (Kennedy and Salama, 2020). Notably, the CD3 co-receptor complex is vital for signal transduction after specific binding of TCR, of which the integrity is considered as the crucial factor for cytotoxic T cell responses to tumor antigens (Fuehrer et al., 2014). CD3E usually participates in encoding the CD3ε chain, one of the major components (γ-, δ-, ε- and ζ-chain) of the CD3 co-receptor complex, whose deficiency will cause the severe combined immunodeficiency (Firtina et al., 2017; Erman et al., 2020). Hart et al. has reported that a reduced cell surface abundance of CD3E could lead to a significant inhibition of T cell killing capacity (Hart et al., 2019). In contrast, the increased expression of CD3E was found markedly related to the effective response in 31 cancer types patients who received anti-PD1 immunotherapy (Gaffney et al., 2019). CD3D has been reported as a potential biomarker for the response to ICIs and prognosis in cancers, including colon cancer and muscle-invasive bladder cancer (Klintman et al., 2016; Shi et al., 2019; Yang et al., 2020). Homozygous mutations in the CD3D gene can lead to markedly abnormal T-cell development, and thus, to early-onset immunodeficiency (Fischer et al., 2005; Gil et al., 2011). Moreover, high expression of CD3E and CD3D gene were reported significantly related to positive OS in CESC (Wang et al., 2019b). LCK, also known as lymphocyte-specific protein tyrosine kinase p56, was found as a key molecule in T cell activation by phosphorylating the TCR/CD3 complex to initiate TCR signaling (Wei et al., 2020). Recent studies showed that the improved LCK activity was contributed to improve the efficacy of chimeric antigen receptors (CARs) immunotherapy in cancers (Gulati et al., 2018; Bommhardt et al., 2019; Suryadevara et al., 2019). In contrast, the inhibited targeted drugs of LCK was reported that could cause the loss of T-cell immune response and result in immunosuppression for patients cancer (Zhao et al., 2008). Therefore, these defined three hub IRGs were expected as immunotherapeutic biomarkers and potential therapeutic targets for CESC patients; this will be the subject of future studies.

The number and proportion of infiltrating immune cells in the TME are recognized as important factors affecting cancer progression and immunotherapy response. To further elucidate the role of the TME associated with this given prognostic signature, the ImmunCellAI database based on ssGSEA algorithm was the first time to be employed to analyze the immune cell infiltrating landscape in CESC patients. We found that most immune cells in low risk groups were more abundant than in high risk group, such as cytotoxic T cell, exhausted T cells, Th1/2 cells, and NK cells, suggesting a more potent immune defense in low risk CESC patients. Additionally, the increased B cells, CD4+ T cell, and CD8+ T cell infiltration indicated that better therapeutic outcomes may be achieved with ICI immunotherapy in low risk groups compared to high risk groups (Liu et al., 2019b; Matsuzaki et al., 2019). Dr. Joy et al. (Hsu et al., 2018) reported that NK cells, in addition to T cells, could enhance the effect of ICI immunotherapy, whereas that efficacy may be weakened due to competition between NK cells and T cells shown in a recent study (Alvarez et al., 2020). In contrast, Tregs and tumor-related macrophages have been reported to be immunosuppressive cells that can form an immunosuppressive atmosphere to facilitate tumor progression by disrupting the adaptive immune response (Milowsky et al., 2016).

Ultimately, to further explore the predictive value of the prognostic signature in ICI immunotherapy, two independent methods, IPS analysis and TIDE algorithm, were applied to calculate the response of CESC patients to ICIs. The results showed that IPS, IPS-CTLA4, IPS-PD1/PD-L1/PD-L2, and IPS/PD1/PD-L1/PD-L2 + CTLA4 scores were significantly increased in this prognostic signature low-risk group. And TIDE algorithm indicated that low risk patients appeared to present with more positive response to anti-ICIs immunotherapy. Both of these founding further support the potential of this given immune-related prognostic signature to determine the immunotherapy sensitivity for CESC patients.

This study represents the first application of WGCNA to identify hub IRGs linked to immune cell infiltration in an effort to develop a prognostic signature for predicting CESC patients prognosis. And it is the first study that employs ImmunCellAI and TIDE algorithm to analyze the immune cell infiltrating landscape and predict immunotherapy sensitivity for CESC, respectively. Compared with individual biomarkers (e.g., PD1 and PDL1) that were more susceptible to interference, the multiple genes signature showed a more reliable stability. In addition, the performance of our three IRGs signature to predict the progression and immune response was better than that in previous study (Liu et al., 2020). Importantly, the versatility of this prognostic model was further verified using external validation, which was less reported in previous study. Nevertheless, some limitations in this work still remain. Firstly, the relative values of gene expression and the difference of FIGO stages between TCGA and GEO database may contribute to the discrepancies in verification groups (e.g., the limited performance in external validation) or in further clinical trails. Secondly, the prognostic signature of CESC patients was developed based on the OS rate, but some external factors such as the TMN degrees were not extensively evaluated due to a lack of relevant data. Thirdly, although bioinformatics tools are helpful to display the interaction of hub IRGs, the external experiments are also important to further elucidate the molecular mechanisms. Finally, TCGA-CESC patients have not received relevant immunotherapy, and the response to ICI treatment was calculated by cutting-edge bioinformatics technologies. Although the potential of this prognostic signature to stratify CESC patients with different immune response was verified reliable by the consistent results of two independent and powerful approaches, a multicenter and large-scale study is still needed to evaluate its practicality in clinical tests and to strengthen its the clinical evidence.

## Conclusion

In the present study, we have identified hub genes related to the immune infiltration in CESC microenvironment and constructed a robust three IRGs (CD3E, CD3D, and LCK) signature to predict the prognosis of CESC patients. Meanwhile, the versatility of the signature was verified by using internal and external validation groups. In the further exploration, we also found that this model had a reliable potential to forecast the sensitivity to ICI immunotherapy for CESC patients, which was able to facilitate personalized counseling for immunotherapy. Further testing of this model in clinical practice will be necessary for prognostic stratification and treatment management in the future.

## Data Availability

Publicly available datasets were analyzed in this study. These data can be found here: TCGA: https://portal.gdc.cancer.gov/; GEO: https://www.ncbi.nlm.nih.gov/geo/.

## Supplementary Material

Supplementary Figure 1 | Association between clinical traits of CESC patients and immune/stromal/ESTIMATE scores. CESC, cervical squamous cell carcinoma; FIGO stage, International Federation of Gynecology and Obstetrics stage.

Supplementary Figure 2 | Sample dendrogram and trait heatmap. Each branch in the image represents one CESC patient and gray below represents missing data.

Supplementary Figure 3 | Analysis of the scale-free fit index for multifarious soft-thresholding powers (β).

Supplementary Figure 4 | LASSO plot of hub IRGs of the prognostic signature. (A) The best lambda was selected at the lowest point of the curve (minimal partial likelihood deviance). LASSO compresses the coefficients of some covariates to 0, leaving thirteen non-zero coefficients. (B) Penalty graph of characteristic thirteen variable coefficients. Three variables (CD3D, CD3E, LCK) with non-zero coefficients were identified at the best lambda, and the feature parameters without information are removed to realize the automatic selection of feature parameters.

Supplementary Figure 5 | The correlation of CD3D, CD3E, LCK expression with tumor purity and immune infiltrating cells.

Supplementary Figure 6 | The correlation of prognostic signature and ESTIMATA ESTIMATE score.

Supplementary Figure 7 | TIDE value of CESC patients to predict the immunotherapy sensitivity. Responder represents positive immunotherapy sensitivity while non-responder represents negative immunotherapy sensitivity. TIDE, Tumor Immune Dysfunction and Exclusion; CESC, cervical squamous cell carcinoma.

Supplementary Table 1 | The clinical/pathological factors between Estimate high score group and low score group.

Supplementary Table 2 | The infiltrating immune cells with the given prognostic signature.

Supplementary Table 3 | The immune functions with the given prognostic signature.
