# Supplementary figures and images for: Exploration of a Robust and Prognostic Immune Related Gene Signature for Cervical Squamous Cell Carcinoma
Source: Front Mol Biosci. 2021 Mar 3;8:625470. doi: 10.3389/fmolb.2021.625470 (PMC7967036; doi:10.3389/fmolb.2021.625470)

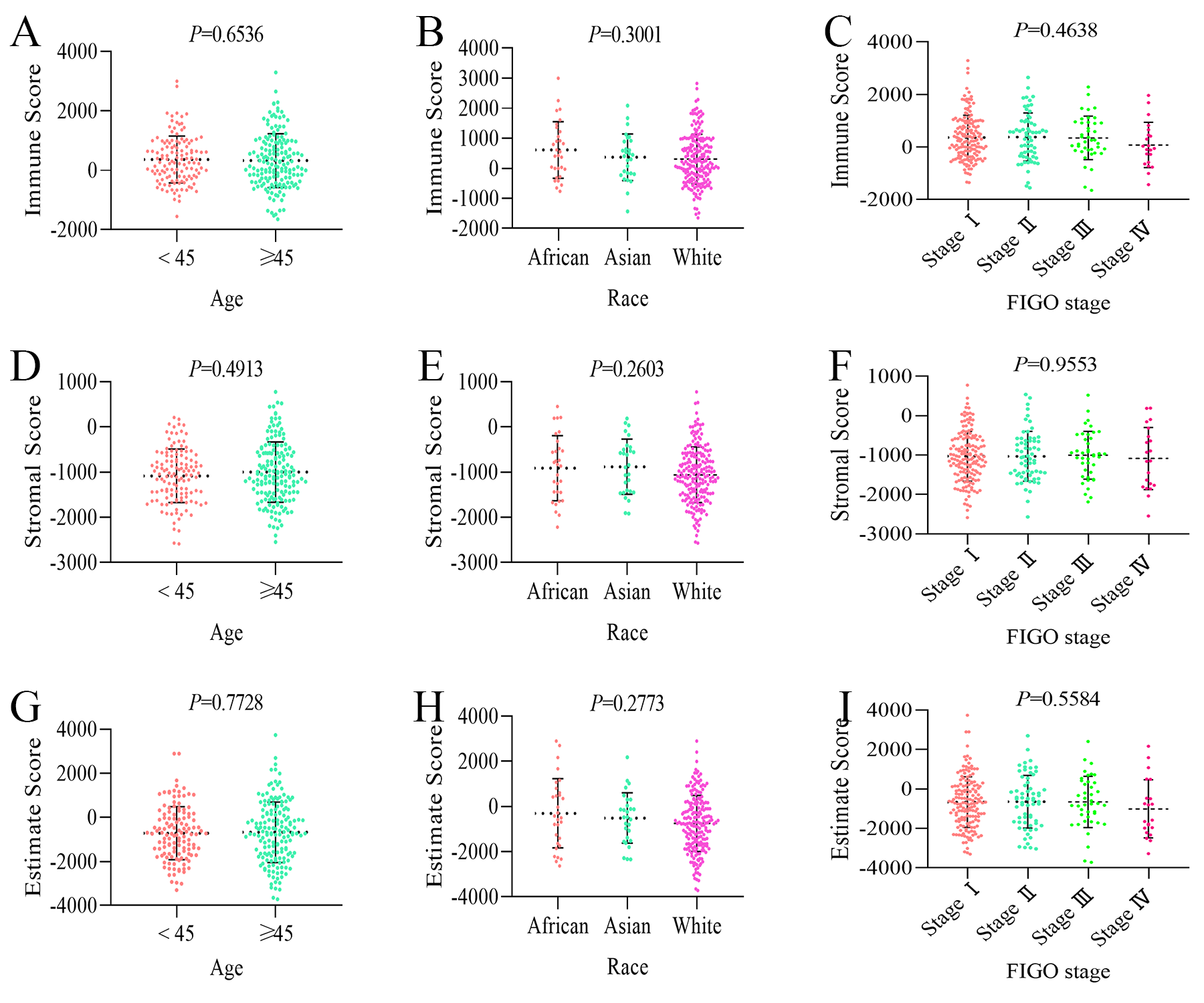

Supplement: Supplementary file 1 [file image1.tif]

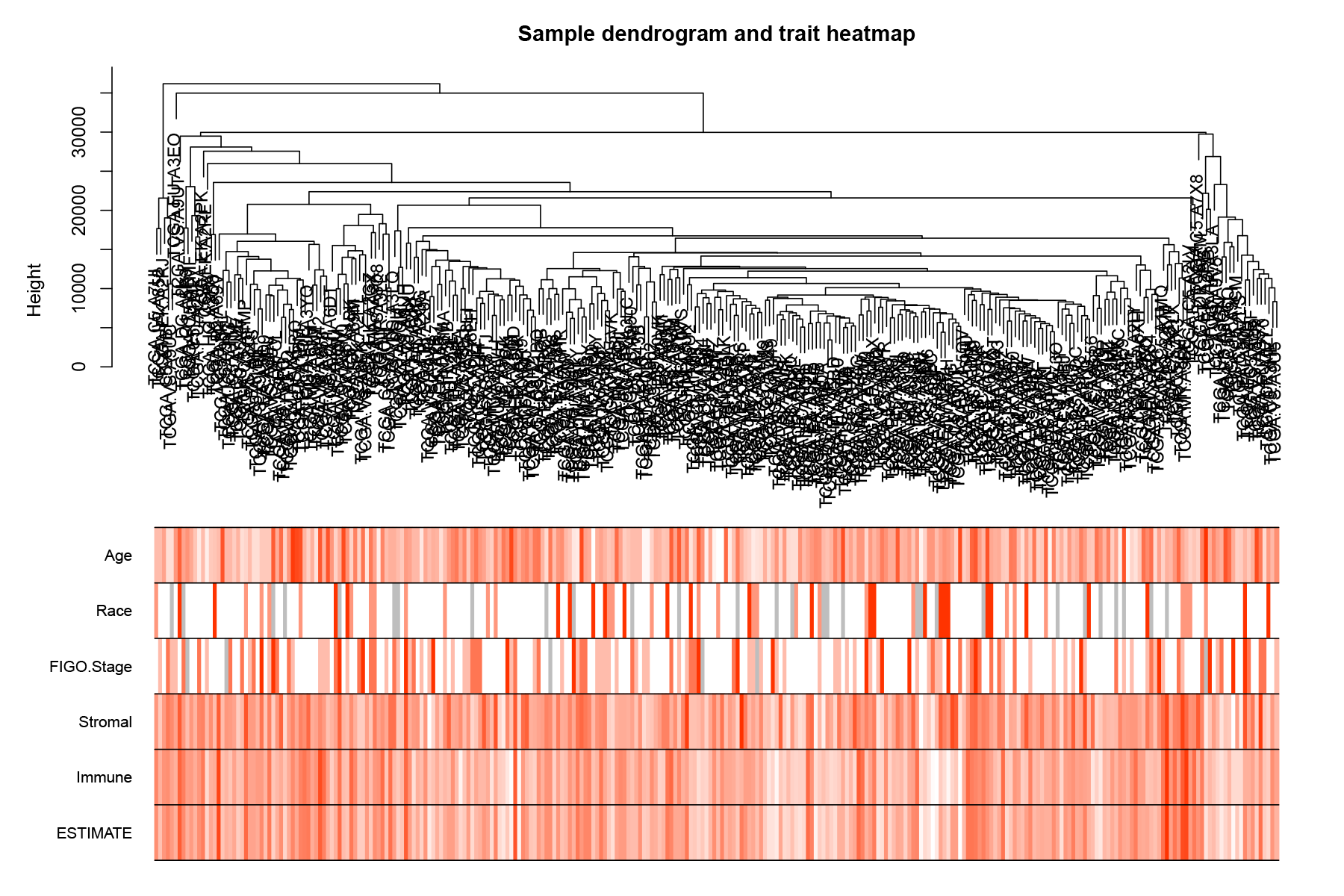

Supplement: Supplementary file 2 [file image2.tif]

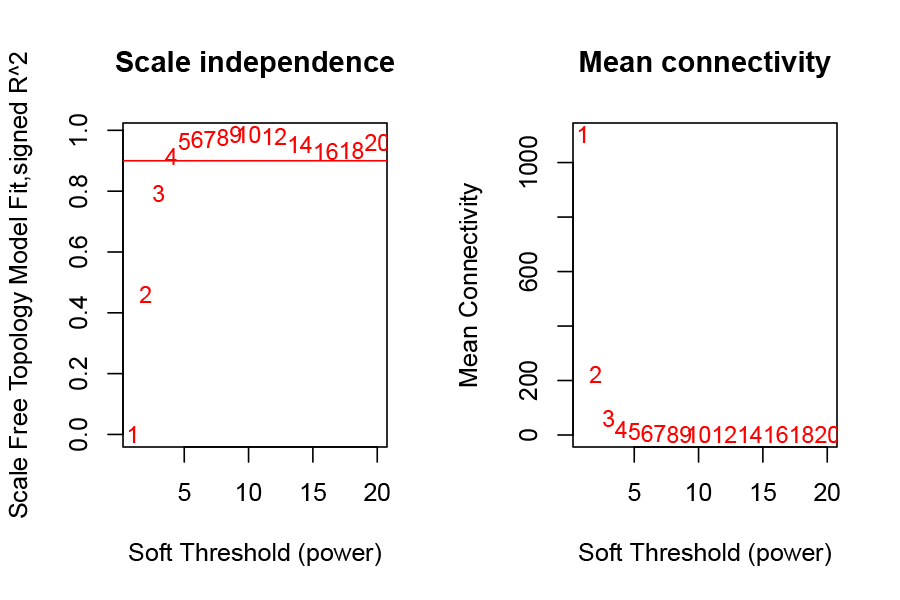

Supplement: Supplementary file 3 [file image3.tif]

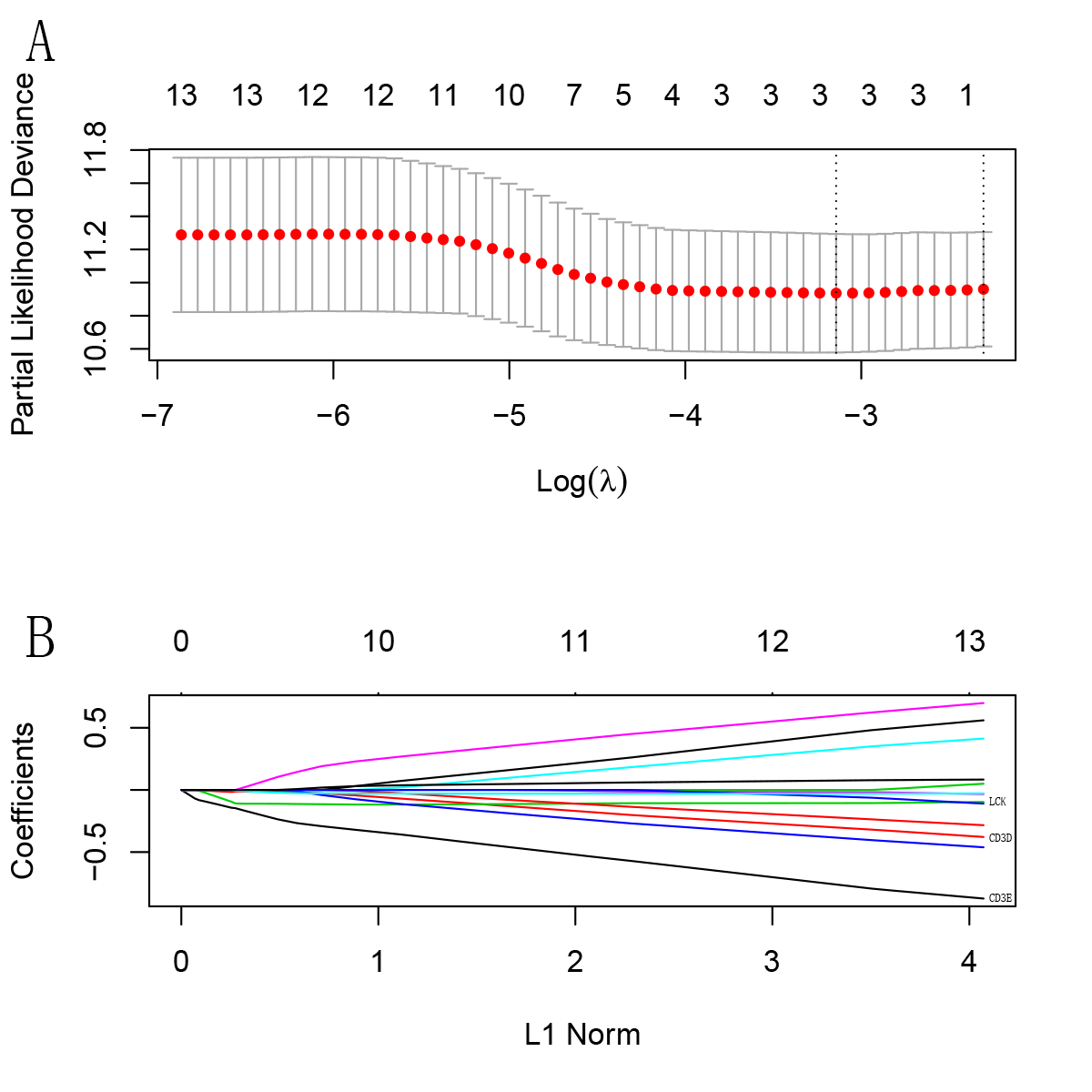

Supplement: Supplementary file 4 [file image4.tif]

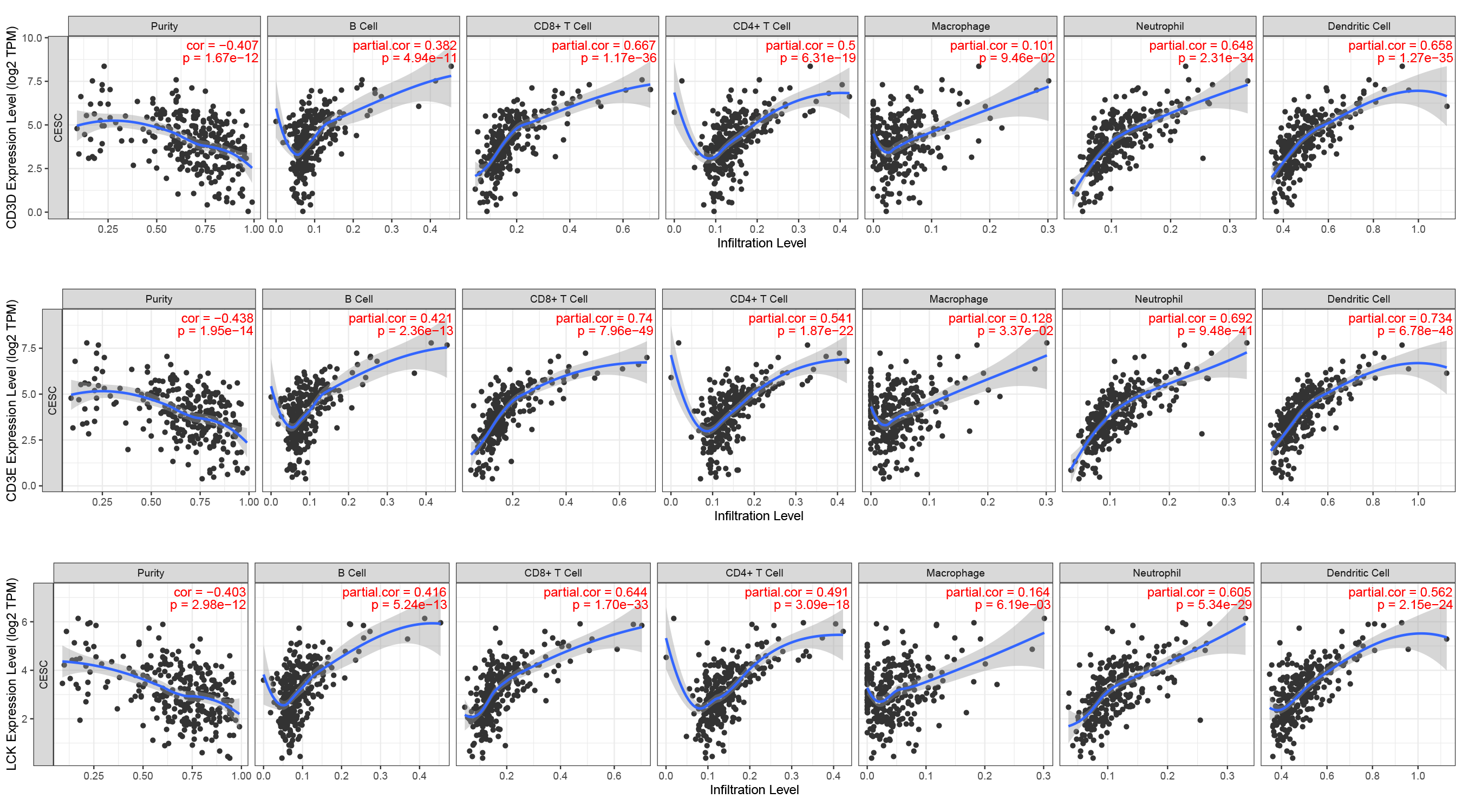

Supplement: Supplementary file 5 [file image5.tif]

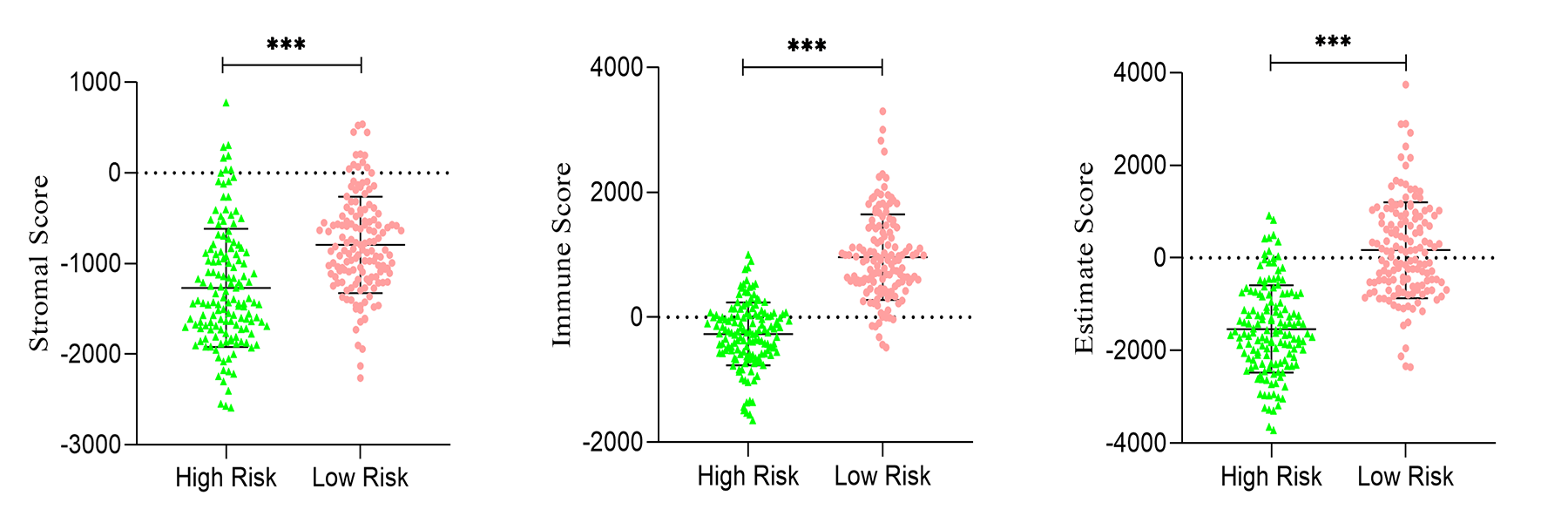

Supplement: Supplementary file 6 [file image6.tif]

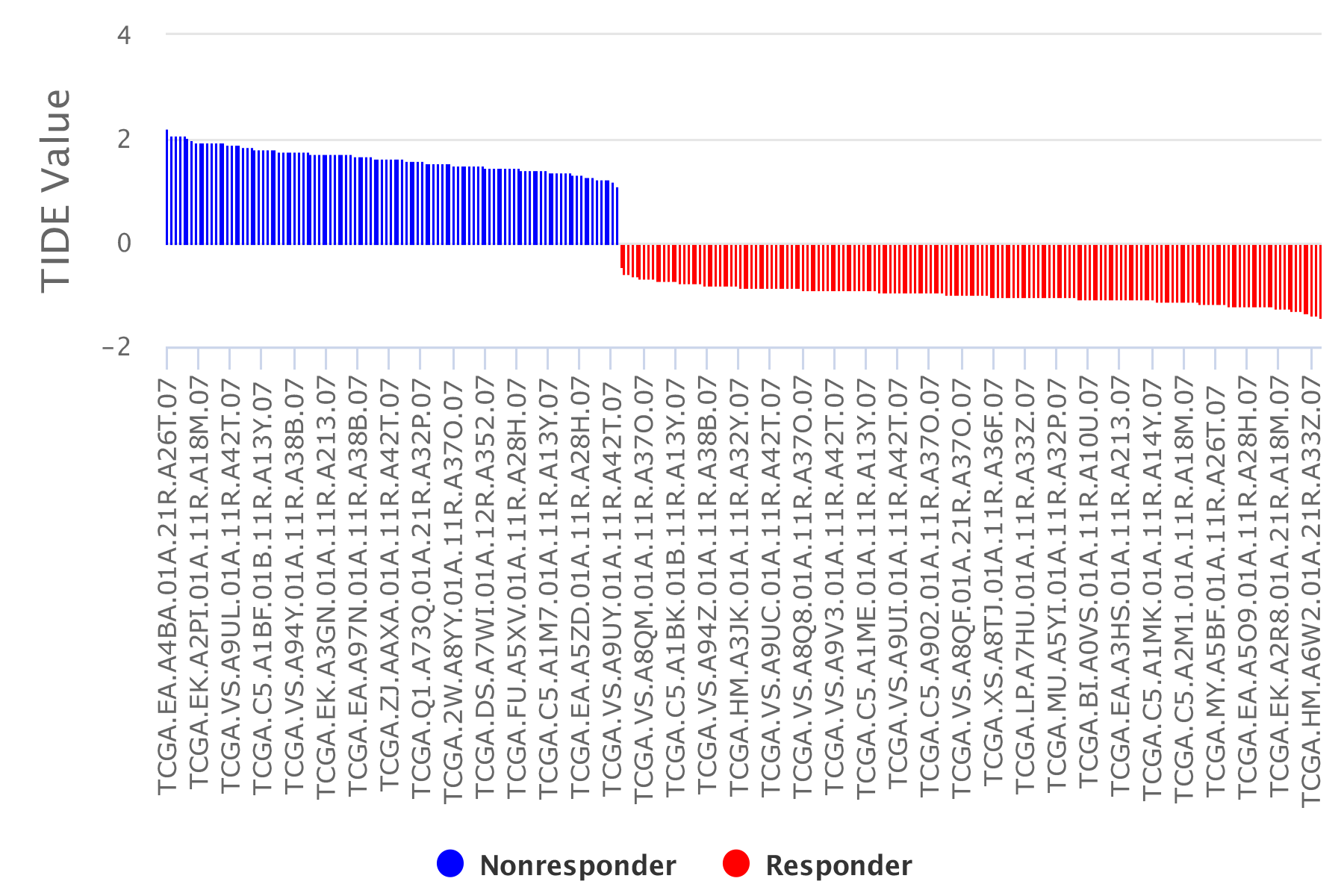

Supplement: Supplementary file 7 [file image7.tif]
